# Association between Immune Markers and Surrogate Markers of Cardiovascular Disease in HIV Positive Patients: A Systematic Review

**DOI:** 10.1371/journal.pone.0169986

**Published:** 2017-01-13

**Authors:** Alinda G. Vos, Annelieke Hulzebosch, Diederick E. Grobbee, Roos E. Barth, Kerstin Klipstein-Grobusch

**Affiliations:** 1 Julius Global Health, The Julius Center for Health Sciences and Primary Care, University Medical Center Utrecht, Utrecht, The Netherlands; 2 Department of Internal Medicine & Infectious Diseases, University Medical Center Utrecht, Utrecht, The Netherlands; 3 School of Clinical Medicine, Faculty of Health Sciences, University of the Witwatersrand, Johannesburg, South Africa; 4 Division of Epidemiology and Biostatistics, School of Public Health, Faculty of Health Sciences, University of the Witwatersrand, Johannesburg, South Africa; University of Bologna, ITALY

## Abstract

**Background:**

HIV infection is associated with an increased risk of cardiovascular disease (CVD). Chronic low-grade immune activation is likely one of the driving mechanisms. This systematic review provides an overview of the evidence addressing the relation between immune markers and surrogate markers of CVD (except CIMT) in HIV infection.

**Methods:**

A systematic search was performed in PubMed, Embase and Cochrane Library identifying all articles from 1996 to April 2015. It addressed the relation between immune markers and surrogate markers of CVD (except Carotid Intima-media Thickness) in HIV-positive adults. Two authors, using predefined criteria, independently conducted the selection of articles, critical appraisal and extraction of the data. Analysis focused on immune markers that were assessed most frequently. The review was conducted according to the PRISMA guideline and performed as part of an overarching review registered with PROSPERO (CRD42014010516).

**Findings:**

Twenty-nine articles were selected, describing 34 immune markers and nine different CVD surrogate outcomes: coronary calcium score (13 times) and flow-mediated dilation (10 times) were used most frequently. Twenty-seven studies had a cross-sectional design. CRP, IL-6 and sVCAM-1 were assessed most frequently. None of the immune markers were clearly associated with any of the surrogate CVD outcomes. No effect estimate could be calculated due to marked heterogeneity in study populations, immune markers, outcomes and statistical approaches.

**Interpretation:**

This review could not identify a clear association between any of the immune markers and surrogate CVD outcomes. This may reflect a true lack of association, or may be explained by heterogeneity across studies and lack of follow-up data. Future research should focus on longitudinal studies measuring a select set of immune markers and surrogate CVD outcomes awaiting the primary outcome of clinical cardiovascular events.

## Background

Highly active antiretroviral therapy (HAART) has markedly increased life expectancy among persons infected with human immunodeficiency virus (HIV). However, it has become clear that patients infected with HIV have an increased risk for developing cardiovascular disease (CVD).[1] Multiple factors contribute to the increased risk for non-AIDS morbidity. An excess burden of traditional risk factors and direct toxic effects of antiretroviral therapy (ART) are the most likely drivers of the pathogenesis and HIV replication has been shown to contribute to the process of atherosclerosis through chronic inflammation and endothelial dysfunction.[2,3] Increased concentrations of immune biomarkers, like C-reactive protein (CRP), IL-6 and D-dimer, indicative of inflammatory processes, have been associated with increased risk of atherosclerosis and mortality in the general population.[4–6] Research in this area has intensified and several studies have addressed the role of immune activation in the occurrence of CVD in HIV infected individuals, identifying a likely association between CRP, IL-6, d-dimer and clinical CVD.[7]

To provide insight in the burden of cardiovascular disease in HIV infected individuals while awaiting results from longitudinal studies, a multitude of surrogate markers for CVD have been used. A recent summary of the current body of evidence regarding potential associations between markers of immune activation and carotid intima media thickness (CIMT) could not draw a clear conclusion due to the heterogeneity in data.[7] This systematic review therefore investigates the relation between markers of immune activation and further surrogate markers of CVD like coronary artery calcium score, flow mediated dilation and pulse wave velocity in HIV infected individuals.

## Methods

This systematic review was conducted according to the guidelines provided by PRISMA[8] (S1 Checklist) and it is part of a larger review registered in the PROSPERO registry for systematic reviews (Registration number CRD42014010516). Results addressing the relation of markers of immune activation, CVD and CIMT were published in January 2016.[7] http://www.crd.york.ac.uk/PROSPERO/display_record.asp?ID=CRD42014010516)

### Search strategy

A systematic search was performed in PubMed, EMBASE and Cochrane Library on April 29^th^ 2015 using terms and synonyms for HIV, immune markers and surrogate markers for CVD other than CIMT, covering all evidence from 1996 onward. Terms were limited to title and abstract (Table 1). The search strategy was an update of the search described in the PROSPERO protocol and published previously.[7]

**Table 1:** Search strategy.

		Search terms
**#1 Domain**	HIV positive patients	HIV
		human immunodeficiency virus
		human immuno deficiency virus
		human immunedeficiency virus
		human immune deficiency virus
		aids
		acquired immunodeficiency syndrome
		acquired immuno deficiency syndrome
		acquired immunedeficiency syndrome
		acquired immune deficiency syndrome
**AND**		
**#2 Determinant**		Inflammatory
		Inflammation
		Inflamm*
		Biomarker
		Biomarkers
		Immune*
**AND**		
**#3 Outcome**	Cardiovascular disease or surrogate markers of cardiovascular disease.	cardiovascular
		CVD
		Myocardial infarction
		mi
		Coronary heart disease
		CHD
		Stroke
		Carotid intima media thickness
		CIMT
		Arterial stiffness
		Flow mediated dilation
		FMD
		PWV
		Pulse wave velocity
		Coronary artery calci*
		CAC
		Ankle brachial index
		ABI

### Selection

**Fig 1:**
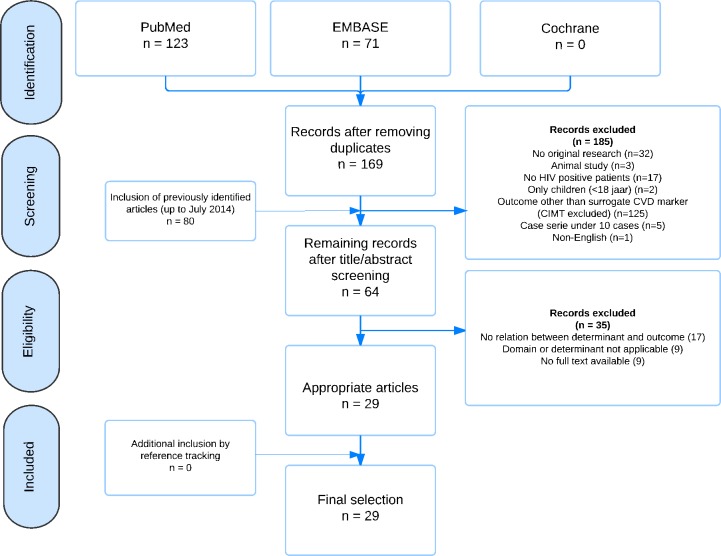
Flowchart inclusion. CIMT: carotid intima media thickness, CVD: cardiovascular disease.

Selection was done in a stepwise process (Fig 1). Firstly, studies were screened on the basis of title and abstract by one author (AH). Studies describing original research including HIV positive patients aged 18 years or above and one of the following surrogate CVD outcomes, were included; CT coronary angiography, coronary artery calcium score (CAC), MRI of blood vessels, arterial inflammation measured by 18FDG PET scan, myocardial perfusion scintigraphy (SPECT), flow mediated dilation (FMD), pulse wave velocity (PWV), pulse wave analysis (PWA), ankle brachial index (ABI) and carotid artery stiffness. Animal studies, articles in a language other than English and case-series describing less than 10 cases were excluded. Secondly, two authors (AH, AV) independently evaluated full-text articles using the following exclusion criteria: only poster abstracts, no relation described between immune marker and no description of an outcome of interest. In case of uncertainty regarding the relation between immune marker and outcome, the corresponding author of the study was contacted once for additional data. Discrepancies in inclusion were discussed in a consensus meeting between two reviewers (AV, AH). A third reviewer (KK) would have been available in case of discrepancies, but agreement could be reached for all inclusions.

### Data extraction and critical appraisal

The following data were extracted: study design and enrollment period, duration of follow up, sample size, country, ethnicity, gender, mean age, duration since HIV diagnosis in years, CD4 count, nadir CD4 count, viral load, BMI, percentage of current smokers, percentage of participants on ART, ART regimen, duration of ART in years, immune markers assessed, use of a stored sample, time of measuring outcome versus immune markers and laboratory methods for immune markers. In case a study used more than one surrogate outcome, only the outcome that was related to immune markers was included in the review.

Selected studies were critically appraised, particularly for the risk of selection-, detection-, and attrition bias. Bias risk was assigned as likely, unlikely, or unclear using an adapted Cochrane Collaboration tool. Data extraction and critical appraisal was performed independently by two authors (AH, AV) using a set format.

### Analysis

Given heterogeneity of studies, a descriptive analysis, grouped by outcome and most frequently assessed immune markers, was conducted. When possible, percentages of common baseline characteristics were calculated.

Surrogate outcomes of CVD were divided in two groups: outcomes using imaging techniques and outcomes assessing arterial stiffness. Imaging techniques were subdivided in two main categories: 1) coronary angiography and coronary artery calcium score, and 2) all other imaging techniques, namely 18FDG PET scan, SPECT and MRI scan of aortic and carotid arteries. Arterial stiffness outcomes were FMD, PWV, PWA, ABI and carotid artery stiffness. Differences in outcome protocols were not taken into account in this review.

Outcome was reported as a positive association, an inverse association, no association or no data. To combine information from various types of studies reporting often more than one risk estimate per outcome, data were reduced by choosing only one effect estimate per immune marker per surrogate CVD outcome per article. To select the effect estimate, the following hierarchical order was used: 1) outcome of multivariable analysis. If not reported, 2) outcome of univariable analysis. If there was no significant outcome in multi-or univariable analysis, the outcome was scored as 3) ‘No association’. If no data were reported, although the methods section specified that the relation between immune marker and outcome has been studied, the outcome was reported as 4) ‘no data’. In this review C-reactive protein (CRP) refers to both the regular CRP measurement and to the high-sensitive CRP assays.

## Results

The updated search identified 169 articles, 64 articles were screened using full text, and 29 articles were finally selected (Fig 1). Articles were excluded if either no relation was described between the immune marker and the surrogate CVD outcome (n = 17), the domain or determinant turned out to be not applicable during full text screening (n = 9) or there was no full text available (n = 9).

### Baseline characteristics

A total of 3,559 HIV positive patients were included, whereof the majority were male (median 80%, interquartile range 59.5–92.0%) (S1 Table); mean age across studies was 45.3 ± 5.4 years, and on average there were almost as many black as white people included in the studies. All except two studies were conducted in the United States of America or in Europe (one study in Australia[9] and in Ethiopia)[10]. Two studies had a longitudinal design with a maximum follow-up duration of 24 weeks.[11,12] Average BMI was 25.1kg/m^2^, (standard deviation (SD) 1.9kg/m^2^) and nearly 40% of all participants were current smokers. Duration of HIV infection ranged between 24 weeks and 16 years. Ten studies[9,13–20] included only participants on ART and three studies included only ART naïve participants.[11,12,21]

CAC score was used as endpoint in 13 studies,[6,13,14,17,22–30] in seven of which a coronary angiography was performed as well.[6,22–27] Five studies used other imaging techniques to assess cardiovascular burden; two studies used 18FDG PET scan[30,31], two used a myocardial perfusion scintigraphy[19,32] and one used an MRI scan of aortic and carotid arteries[33]. Arterial stiffness was assessed in 13 studies[9–12,15–18,20,21,34–36]; 10 studies used FMD[9–12,15–17,20,21,34], two PWV[10,20], one PWA[18], one ABI[35] and one used carotid artery stiffness[36].

### Critical appraisal

**Fig 2:**
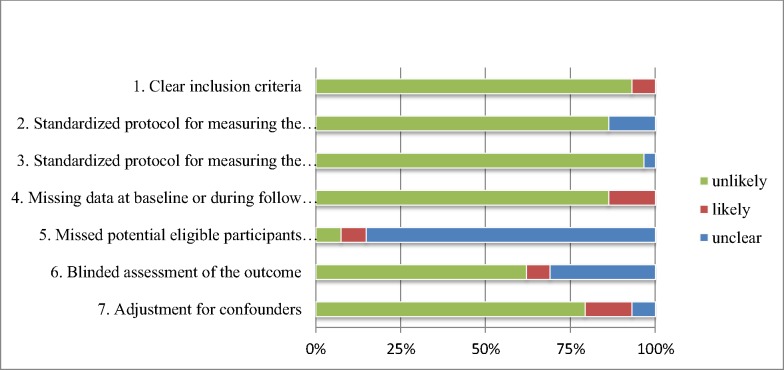
Risk of bias assessment.

All studies were critically appraised on eight items; clarity of inclusion criteria; moment of inclusion; standardization of measurement of determinant and outcome; missing data at baseline or follow-up; missing data on potential eligible participants; and blinding and adjustment for confounders (S2 Table). A risk of bias summary is presented in Fig 2. The criterion ‘homogeneous moment of inclusion’ is not incorporated in the figure, since it could not be categorized as ‘likely’ or ‘unlikely’ due to the different aspects that were covered. Risk of bias was low in general, except for the missed potential eligible participants. Although most studies had a standardized procedure for measuring immune markers and outcomes, these procedures were different between studies.

### Overall outcome

**Fig 3:**
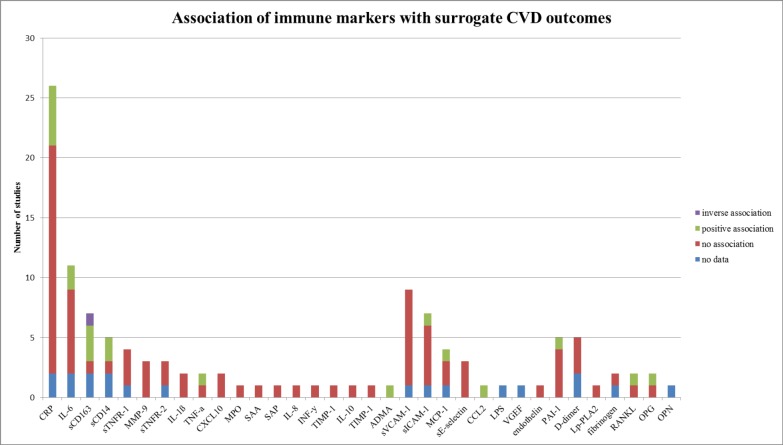
Association of immune markers with surrogate CVD outcomes. CVD: cardiovascular disease.

Fig 3 provides an overview of the number of studies that assessed any immune marker in relation to the surrogate CVD outcomes. CRP, IL-6 and sVCAM-1 were assessed most frequently. None of the markers were clearly associated with surrogate markers of CVD. Only CRP and sCD163 were at least three times positively associated: CRP 5 times (out of 26 studies),[10,13,18,22,29] of which three times in univariable analysis,[13,22,29] sCD163 three times (out of 7 studies),[6,27,30] of which two times in univariable analysis.[6,30]

### Imaging

#### CT coronary angiography and coronary calcium score

**Fig 4:**
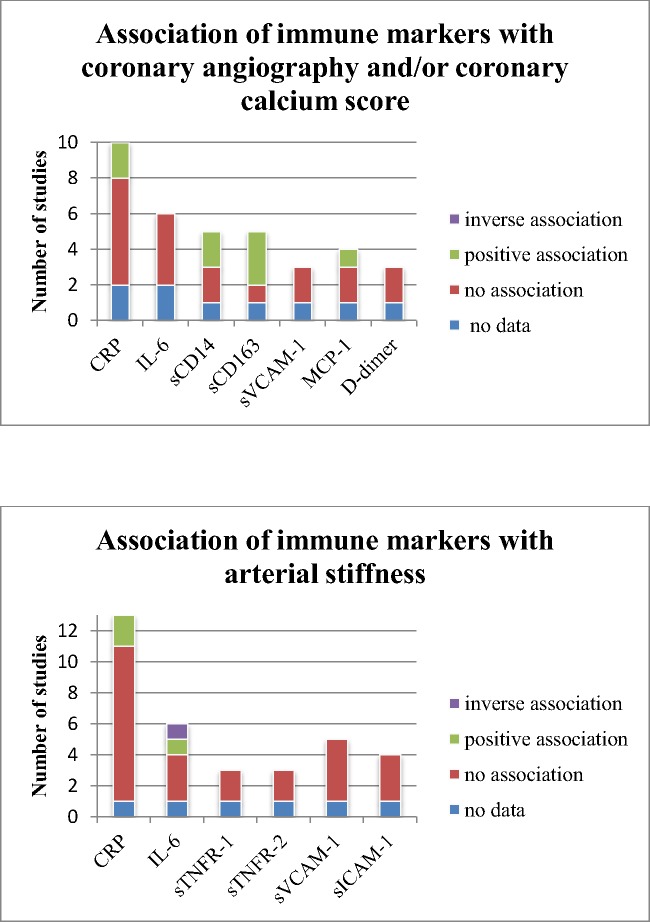
Association of immune markers with coronary angiography, coronary calcium score and arterial stiffness.

Six Smarkers were evaluated three or more times in relation to coronary calcium score or coronary angiography (Fig 4A). CRP was assessed 10 times,[6,13,14,17,22–26,29] of which two studies showed a significant, but weak correlation with severity of obstruction on angiography (Rho 0.29, p 0.038)[22] and with CAC (Rho value 0.16, p 0.003)[29]. sCD163 was positively related in three out of five studies; two times to non-calcified plaque[6,23] and one time to coronary calcium score and coronary artery stenosis of more than 50%.[27] However, the association with non-calcified plaque disappeared in multivariable analysis in one out of two studies.[6] sCD14 showed a significant association in two out of five studies. A high level of sCD14 compared to a low level of sCD14 was associated with an odds ratio of 3.3 (95% CI 1.1–9.7) for coronary artery stenosis,[27] and levels of sCD14 were related to the presence of coronary artery calcium in multivariable analysis.[13] MCP-1 was borderline significantly related to severity of plaque (Rho value 0.23, p 0.047) and weakly related to the Agatston Calcium score (Rho value 0.27, p 0.02) in one study.[26]

#### Other imaging techniques

CRP was assessed in four studies using 18FDG PET scan[30,31] or SPECT scan as outcome[19,32]; no study showed a significant association. None of the other markers were assessed more than three times. sCD163 was assessed twice in relation to PET scan outcomes, both studies showed a significant association (association with FDG uptake in the descending aorta Rho value -0.517, p 0.007] and with Target Background Ration, Rho value 0.31, p 0.04).[30]

### Arterial stiffness measurement

Six markers were evaluated three times or more in relation to arterial stiffness (Fig 4B). CRP was positively associated with PWV in multivariate analysis (parameter estimate 0.0209, p 0.01),[10] and mean levels of CRP were significantly higher in individuals with definite peripheral artery disease compared to participants with normal ABI (8.5 versus 7.2 mg/L).[11,12,35] Levels of IL-6 corresponded with the lowest quartile of FMD (OR 1.17, 95% CI 1.01–1.35, p 0.04) and IL-6 was the best predictor of FMD in a multivariable linear model.[16] On the other hand, Stein et al[21] found an inverse relation in univariate analysis; higher IL-6 levels were associated with higher FMD (indicative of a lower CVD risk) and lower levels of IL-6 were associated with increase in brachial diameter (indicative of a higher CVD risk). None of the other markers were significantly associated with arterial stiffness. Three studies using FMD as outcome included only ART naïve individuals.[11,12,21] No association with CRP was found (assessed in all three studies), and associations with IL-6 (assessed in two studies) showed contradictory results.

### Composite endpoint for vascular disease

Longenecker et al.[13] used a composite endpoint for vascular disease consisting of CAC score >0, endothelial dysfunction according to FMD and carotid disease. They found that CRP, sCD14 and fibrinogen were all significantly associated with vascular disease.[13]

## Discussion

A large number of immune markers have been investigated for predictive value in relation to surrogate markers of cardiovascular disease in HIV positive patients. CRP, IL-6 and sVCAM-1 were addressed most frequently; neither CRP, IL-6, sVCAM-1 nor any of the other markers showed a clear relation with any of the surrogate CVD markers.

These results complement the findings of a recent review assessing immune marker choice in relation to CVD or CIMT. CRP, IL-6 and s-VCAM-1 were also the most frequently assessed markers in relation to CIMT, and the lack of a clear relation between any of these immune markers and surrogate CVD outcomes that is currently found is in line with the findings in relation to CIMT.[7]

Although there is ample evidence that levels of CRP[37,38] and IL-6[39,40] are related to CVD both in the general population as well as in the HIV-infected population,[5,41–43] the current review does not indicate any consistent relation of CRP and IL-6 to coronary calcium score, coronary stenosis, SPECT, 18FDG PET scan or measurements of arterial stiffness.

Although the prognostic role of s-VCAM-1 levels in the prediction of CVD in not as clear, an association with signs of endothelial damage (FMD, PWV) or inflammation (MRI, PET) was to be expected as s-VCAM-1 is expressed on the endothelial surface in case of endothelial inflammation.[44] However, no single positive association with any of the surrogate CVD outcomes was detected in this review.

The two possible explanations why this review did not show an association between any of the markers and surrogate CVD outcomes could be that either there is no association, or associations are not yet clear due to methodological and qualitative constraints in the available evidence.

The first explanation, no association, could be due to the fact that, despite surrogate outcomes and overt CVD being clearly related,[45–53] and CRP, IL-6 and overt CVD having a clear relation, associations between markers of immune activation and surrogate markers of CVD may be weak.

The second explanation, lack of an association due to methodological and qualitative constraints, could be due to a marked heterogeneity in choice of both immune markers, surrogate outcomes and ways of analysis (effect estimates vary from correlations, mean markers levels, odds ratio’s and multivariable analysis). In addition, studies were not primarily designed or powered to detect an association between immune markers and outcome and study designs were mainly cross-sectional (27 out of 29 studies). Follow-up time of the two prospective studies was too short to detect significant vascular alterations and all studies included a relatively young population (average age 45 years), in which atherosclerotic vascular changes and–burden are expected to be low.

Finally, possible associations might be masked by heterogeneity in patient populations, duration of HIV diagnosis, use and duration of antiretroviral therapy (ART), viral suppression rates and CD4 counts, as they differ substantially in, and between, studies. All these factors have been previously reported to influence both the inflammatory response and the risk of cardiovascular morbidity and mortality.[53–55] Moreover, statin use is not taken into account in most of the included studies, whereas recent evidence suggests that statin use can improve surrogate cardiovascular outcomes.[56,57]

### Strengths and limitations

This systematic review synthesizes the available information on immune-markers in relation to surrogate markers of CVD (except CIMT) in HIV-infected patients from 1996 up to April 2015. The main focus of this review was to provide an overview of which immune markers were assessed, how frequently these markers were assessed and which surrogate CVD outcomes were chosen, as well as to summarize the current evidence on the relation between immune markers and outcomes.

The most frequently assessed immune markers (CRP and IL-6) have been shown to be related to CVD morbidity and mortality in both the general population and in the HIV-infected population, and surrogate CVD outcomes have been shown to be related to CVD in the general population. This is the first time that the relation between immune markers and surrogate CVD outcomes is addressed in a systematic way in the context of HIV infection. The major strength of this review is that it contributes to a clear, global understanding of existing knowledge regarding immune markers and surrogate CVD outcomes.

Limitations to be considered include that only one outcome per marker per article was considered which might have resulted in an overly optimistic impression of associations as negative outcomes are underreported; that positive outcomes include univariable associations which might in part be confounded; and that outcomes were grouped in categories (CT-angiography/CAC, other imaging techniques and arterial stiffness) which might have resulted in specific associations (to specific parts of an outcome) being overlooked.

## Conclusion

This review provides an overview of the current literature regarding the association between immune markers and surrogate markers of cardiovascular disease other than CIMT in HIV-positive individuals. Most frequently assessed immune markers were CRP, IL-6 and s-VCAM-1; most frequently assessed surrogate markers for CVD were CAC and FMD.

No relation between any of the immune markers and any of the surrogate outcomes could be detected. This may be due to the cross-sectional design, heterogeneity in patient populations, the variety in immune marker choice and surrogate CVD outcomes. The search for the association of immune markers in relation to surrogate CVD outcomes in a cross-sectional study design should be reconsidered.

Future research should focus on longitudinal studies measuring immune markers and surrogate CVD outcomes awaiting the primary outcome of clinical cardiovascular events.

## Supporting Information

S1 Checklist PRISMA 2009 Checklist. (DOC)

S1 Table Baseline table. (DOCX)

S2 Table Critical appraisal. ND: no data.
(DOCX)
