# Supplementary material for: Association between Immune Markers and Surrogate Markers of Cardiovascular Disease in HIV Positive Patients: A Systematic Review
Source: PLoS One. 2017 Jan 13;12(1):e0169986. doi: 10.1371/journal.pone.0169986 (PMC5234789; doi:10.1371/journal.pone.0169986)
Supplement: S1 Table — (DOCX) [file pone.0169986.s002.docx]

| **Author** | | | **Title** | | | | | | | | **Design, enrollment period and follow-up duration** | | | | | **HIV+**  **Patients (n)** | | | **Country** | | | **Ethnicity** | | | **% ♂** | | **Age** | | **Years since HIV diagnosis** | | **CD4 count** | | | **Nadir CD4 count** | | **Viral load (log copies/mL)** | | **BMI (kg/m2)** | | | | **Current smokers** | | **ART** | | **Tx regimen** | | | **Duration of ART (years)** | **Markers assessed** | | | | **Stored sample used** | **Time of outcome - biomarker** | **Methods** |
| --- | --- | --- | --- | --- | --- | --- | --- | --- | --- | --- | --- | --- | --- | --- | --- | --- | --- | --- | --- | --- | --- | --- | --- | --- | --- | --- | --- | --- | --- | --- | --- | --- | --- | --- | --- | --- | --- | --- | --- | --- | --- | --- | --- | --- | --- | --- | --- | --- | --- | --- | --- | --- | --- | --- | --- | --- |
| **CT coronary angiography and coronary artery calcium score (CAC)** | | | | | | | | | | | | | | | |  | | |  | | |  | | |  | |  | |  | |  | | |  | |  | |  | | | |  | |  | |  | | |  |  | | | |  |  |  |
| Burdo,T. H.;Lo,J.;Abbara,S.; | | | Soluble CD163, a novel marker of activated macrophages, is elevated and associated with noncalcified coronary plaque in HIV-infected patients | | | | | | | | Cross-sectional | | | | | 102 | | | USA | | | white 63%, black 22%, hispanic 12%, Asian 1%, Native American 3% | | | 100 | | 46.6 ± 6.4 | | 13.8 ± 6.4 | | 530 ± 287 | | | 202 ± 173 | | <50 (<50 to <50) 81% <50 | | 26.3 ± 4.7 | | | | 41% | | 95% | | 52% PI, 92% NRTI, 47% NNRTI | | | 7.2 ± 5.0 | sCD163, LPS, sCD14, Osteopontin, MCP-1, hsIL-6, CRP, d-dimer | | | | no | = | ELISA, LAL assay (LPS), immunoturbidometry (d-dimer) |
| D'Ettorre,G.;Ceccarelli,G.;Francone,M.; | | | High prevalence of coronary stenosis detected by Coronary CT angiography in asymptomatic HIV-infected subjects with low cardiovascular risk | | | | | | | | Cross-sectional | | | | | 55 | | | Italy | | | nd | | | 86 | | 47.6 ± 8.7 | | 9.5 ± 5.5 | | 493 ± 223 | | | 193 ± 126 | | 2.3 ± 1.1, 74.5% <50 | | 22.2 ± 2.5 | | | | nd | | 89.1 | | nd | | | 9.4±5.0 | hsCRP | | | | nd | ? | ELISA |
| Fitch,Kv;Srinivasa,S.;Abbara,S.; | | | Noncalcified coronary atherosclerotic plaque and immune activation in HIV-infected women | | | | | | | | Cross-sectional | | | | | 60 | | | USA | | | white 25%, nonwhite 75% | | | - | | 47 ± 7 | | 15 ± 6 | | 597 ± 297 | | | 191 ± 160 | | 4.1 ± 0.9. 84% undetectable viral load | | 28 ± 6 | | | | 50% | | 98 | | 58% PI, 92% NRTI, 17% NNRTI | | | 8 ± 5 | hsCRP, hsIL-6, sC163, sCD14, CXCL10, MCP-1 | | | | no | = | ELISA |
| Hwang,J. J.;Wei,J.;Abbara,S.; | | | Receptor activator of nuclear factor-kappaB ligand (RANKL) and its relationship to coronary atherosclerosis in HIV patients | | | | | | | | Cross-sectional | | | | | 78 | | | USA | | | white 68%, black 18%, asian 1%, hispanic 9%, native American 4% | | | 100 | | 46.5 ± 6.5 | | 13.5± 6.1 | | 523 ± 282 | | | 169 (54 - 263) | | <50 (<50 to <50) 81% <50 | | 26.1 ± 4.3 | | | | 35% | | 95 | | 53% PI | | | 7.1 ± 4.6 | RANKL, osteoprotegerin, CRP | | | | nd | ? | ELISA |
| Lai,S.;Bartlett,J.;Lai,H.; | | | Long-term combination antiretroviral therapy is associated with the risk of coronary plaques in African Americans with HIV infection | | | | | | | | Cross-sectional, Aug 2003 to Dec 2007 | | | | | 176 | | | USA | | | African American 100% | | | 63 | | 44.2 (39.9-47.3) | | nd | | 344 (177-498) | | | nd | | 581 (30-14789) cp/mL | | 23.8 (21.5-27.3) | | | | 86.4% | | nd | | nd | | | In months: NRTI's 0 (0-12), NNRTIs 0 (0-0), PIs 0 (0-12), All 3 (0-24) | hsCRP | | | | nd | = | nd |
| Lo,J.;Abbara,S.;Shturman,L. | | | Increased prevalence of subclinical coronary atherosclerosis detected by coronary computed tomography angiography in HIV-infected men | | | | | | | | Cross-sectional | | | | | 78 | | | USA | | | white 68%, black 18%, asian 1%, hispanic 9%, native American 4% | | | 100 | | 46.5 ± 6.5 | | 13.5 ± 6.1 | | 523 ± 282 | | | 169 (54 - 263) | | <50 (<50 to <50) 81% <50 | | 26.1 ± 4.3 | | | | 35% | | 95 | | 53% PI, 91% NRTI, 49% NNRTI | | | 7.1 ± 4.6 | MCP-1, CRP, IL-6 | | | | nd | ? | ELISA. No data for IL-6 |
| McKibben,R A.;Margolick,J B.;Grinspoon,S; | | | Elevated levels of monocyte activation markers are associated with subclinical atherosclerosis in men with and those without HIV infection | | | | | | | | Cross sectional. Jan 2010-Aug 2013 | | | | | 566 | | | USA | | | white 51.9%, African American 34.3%, Hispanic/other 13.8% | | | 100 | | 53.0 ± 6.5 | | nd | | 599 (426-751) | | | 251 (144-335) | | 81% <50 copies/mL, rest: 537 (131-12200) copies/mL | | 26.2 ± 4.5 | | | | 31.3% | | 95.9 | | nd | | | 12.3 (8.8-14.1) | sCD163, sCD14, CCL2 | | | | yes | = | ELISA for sCD163 and sCD14, Luminex-based singleplex cytokine panel for CCL2 |
| **Coronary calcium score (CAC)** | | | | | | | | | | | | | | | |  | | |  | | |  | | |  | |  | |  | |  | | |  | |  | |  | | | |  | |  | |  | | |  |  | | | |  |  |  |
| Jang,J. J.;Berkheimer,S. B.;Merchant,M.; | | | Asymmetric dimethylarginine and coronary artery calcium scores are increased in patients infected with human immunodeficiency virus | | | | | | | | Case control, from Jan 2007 to Dec 2008 | | | | | 37 | | | USA | | | nd | | | 73 | | 45.0 ± 8.0 | | nd | | nd | | | nd | | nd | | 27.5 ± 7.5 | | | | past or current: 54.0% | | nd | | nd | | | nd | ADMA | | | | nd | ? | ADMA by chromotography |
| Longenecker,C. T.;Jiang,Y.;Orringer,C. E.; | | | Soluble CD14 is independently associated with coronary calcification and extent of subclinical vascular disease in treated HIV infection | | | | | | | | Cross-sectional, substudy of SATURN-HIV trial | | | | | 147 | | | USA | | | African american 69% | | | 78 | | 46 (40-53) | | 12 (6.2-18) | | 613 (425-853) | | | 179 (86-298) | | 70% <48 copies/mL | | 27 (23-30) | | | | 63% | | 100% | | 49% PI, 5% ZDV or D4T, 5% abacavir | | | 5.3 (3.2-9.8) | sCD14, sCD163, hsCRP, IL-6, sTNFR-I, sVCAM-1, D-dimer, fibrinogen, OPG, RANKL. | | | | nd | = | ELISA, nephelometer (hsCRP and fibrinogen), immunoturbidometric assay (d-dimer), singleplex immunoassay (RANKL). |
| Mangili,A.;Ahmad,R.;Wolfert,R. L. | | | Lipoprotein-associated phospholipase A2, a novel cardiovascular inflammatory marker, in HIV-infected patients | | | | | | | | Cross-sectional, CAC computed from Jan 2002 to Dec 2003 | | | | | LpPLA2 mass <235: 84 | | | USA | | | 76% non white | | | 69 | | 44 ± 7 | | 10.2 ± 4.9 | | 385 ± 256 | | | nd | | 3.6 ± 1.2 (36% undetectable) | | 28 ± 6 | | | | 54% | | 64% | | 36% on PI | | | 25 ± 25 months | Lp-PLA2 mass, CRP | | | | yes | unclear | ELISA, immunoturbidometry (CRP) |
|  |  |  |  |  |  |  |  |  |  |  |  |  |  |  |  | LpPLA2 mass >235: 257 | | |  |  |  | 39% non white | | | 76 | | 44 ± 7 | | 9.6 ± 4.8 | | 470 ± 308 | | |  |  | 2.9 ± 1.0 (62% undetactable) | | 26 ± 5 | | | | 47% | | 77% | | 47% on PI | | | 33 ± 26 months |  |  |  |  |  |  |  |
| Shikuma,C. M.;Barbour,J. D.;Ndhlovu,L. C.; | | | Plasma monocyte chemoattractant protein-1 and tumor necrosis factor-(alpha) levels predict the presence of coronary artery calcium in HIV-infected individuals independent of traditional cardiovascular risk factors | | | | | | | | Cross sectional. Substudy of Hawaii Aging with HIV Cardiovascular (HAHC-CVD) study | | | | | 130 | | | USA | | | 60% white, 3.1% African American, 2.3% Native american/alaskan, 12.3% Native hawaiian/pacific islanders, 7.7% asian, 14.6% mixed | | | 88 | | 51 (46-57) | | nd | | 492 (341-635) | | | 154 (30-250) | | 86.9%<50 copies/ml | | 25.8 (23.9-28.3) | | | | 22.3% (of patients with clinical CVD) | | 100% | | nd | | | nd | sE-selectin, sVCAM-1, sICAM-1, MMP-9, MPO, tPAI-1, CRP, SAA, SAP, IL-1B, IL-6, IL-8, IL-10, TNF-a, MCP-1, VEGF, IFN-y | | | | yes | unclear | Multiplex (Milliplex Human Cardiovascular disease panels) |
| **Flow mediated dilation (FMD)** | | | | | | | | | | | | | | | |  | | |  | | |  | | |  | |  | |  | |  | | |  | |  | |  | | | |  | |  | |  | | |  |  | | | |  |  |  |
| Gupta,S. K.;Mi,D.;Dube,M. P.; | | | Pentoxifylline, inflammation, and endothelial function in HIV-infected persons: a randomized, placebo-controlled trial | | | | | | | | Randomized double blinded placebo controlled trial  FU 8 weeks | | | | | Placebo: 13 | | | USA | | | 62% black race | | | 85 | | 34 ± 10.9 | | nd | | 583 ± 175 | | | nd | | 4.0 ± 1.2 | | 27.7 ± 5.9 | | | | 38% | | 0 | | na | | | na | hsCRP, IL-6, sTNFRI, sTNFRII, MCP-1, TIMP-1, IP-10, PAI-1 Ag, sVCAM-1 | | | | yes | = | hsCRP, IL-6, IP-10, lipids and insulin: serum. MCP-1, sTNFRI and II, sVCAM-1 and TIMP-1: EDTA plasma and PAI-1 Ag : citrated plasma. No information on technique. |
|  |  |  |  |  |  |  |  |  |  |  |  |  |  |  |  | Pentoxifylline: 13 | | |  |  |  | 62% black race | | | 62 | | 40 ± 11.6 | |  |  | 524 + 165 | | |  |  | 4.0 ± 0.7 | | 26.1 ± 4.6 | | | | 46% | | 0 | |  |  |  |  |  |  |  |  |  |  |  |
| Hileman,C. O.;Longenecker,C. T.;Carman,T. L | | | Elevated D-dimer is independently associated with endothelial dysfunction: a cross-sectional study in HIV-infected adults on antiretroviral therapy | | | | | | | | Cross-sectional | | | | | 98 | | | USA | | | African american 52%, Caucasian 42% | | | 88 | | 47.5 (43-52) | | 11.3 (7.2-16.2) | | 579 (431-789) | | | 130 (32-238) | | all <400 copies/ml | | 26 (23.3-30) | | | | 47% | | 100 | | PI 52%, NNRTI 48% | | | nd | IL-6, sTNFR-I, sTNFR-II, hsCRP, sICAM-1, sVCAM-1, fibrinogen, d-dimer | | | | yes | = | ELISA, immunonephelometry (hsCRP, fibrinogen), immunoturbidometry (d-dimer) |
| Masia,M.;Padilla,S.;Garcia,N.; | | | Endothelial function is impaired in HIV-infected patients with lipodystrophy | | | | | | | | Cross-sectional, Jan - June 2008 | | | | | No lipodystrophy: 55 | | | Spain | | | nd | | | 80 | | 46.0 (36.3-50.3) | | nd | | 445 (337.5-645.0) | | | nd | | 75% <50 copies/ml | | 23.7 (21.8-27.3) | | | | 61.8% | | 100% | | 25.5% NN analogue based regimen, 69.1% PI based regimen, 32.7% abacavir containing regimen, 16.4% thymidine analogue containing regimen | | | 7 (4-9.25) | sICAM-1, sVCAM-1, sE-selectin, hsCRP, IL-6, TNF-a, PAI-1 | | | | yes | = | ELISA, IMMULITE 2000 analyzer (CRP) |
|  |  |  |  |  |  |  |  |  |  |  |  |  |  |  |  | Lipodystrophy: 55 | | |  |  |  |  |  |  | 82 | | 45.5 (41.9-52.2) | |  |  | 550 (347.5-805.0) | | |  |  | 76.7% <50 copies/ml | | 24.6 (21.7-27.2) | | | | 52.7% | |  |  | 51.9% NN analogue based regimen, 44.4% PI based regimen, 36.4% abacavir containing regimen, 5.5% thymidine analogue containing regimen | | | 11 (9-13) |  |  |  |  |  |  |  |
| Nolan,D.;Watts,G. F.;Herrmann,S. E.; | | | Endothelial function in HIV-infected patients receiving protease inhibitor therapy: does immune competence affect cardiovascular risk? | | | | | | | | Cross-sectional | | | | | 24 | | | Australia | | | nd | | | 100 | | 42.6 ± 9.5 | | nd | | 469 ± 240 | | | 134 ± 157 | | 75% <50 copies/ml (mean: log 3.97 ± 1.1 copies/mL) | | 24.1 ± 2.7 | | | | 41.6% | | 100% | | 100% PI | | | 30.5 ± 4.9 months (PI) | CRP | | | | no | = | Immunonephelometry |
| Solages,A.;Vita,J. A.;Thornton,D. J.; | | | Endothelial function in HIV-infected persons | | | | | | | | Cross-sectional | | | | | 75 | | | USA | | | Black 56%, white 21%, hispanic 20% | | | 56 | | 44.2 ± 8.4 | | nd | | nd | | | 270 ± 250 | | 90 | | 26.6 ± 4.5 | | | | 63% | | 84% | | 43% PI, 57% non PI regimen | | | nd | hsCRP | | | | nd | = | nd |
| Stein,Jh;Brown,Tt;Ribaudo,Hj; | | | Ultrasonographic measures of cardiovascular disease risk in antiretroviral treatment-naive individuals with HIV infection | | | | | | | | Cross-sectional | | | | | 331 | | | USA | | | 44% white, 32% black, 20% hispanic | | | 89 | | 36 (28-45) | | 0.5 (0.2-2.6) | | 349 (207-455) | | | nd | | 4.5 (4.0-5.1) | | 25 (22-28) | | | | 38% | | 0% | | na | | | na | hsCRP, IL-6 | | | | yes | unclear | Nephelometry (hsCR)), ELISA (IL-6) |
| Torriani,F. J.;Komarow,L.;Parker,R. A.; | | | Endothelial function in human immunodeficiency virus-infected antiretroviral-naive subjects before and after starting potent antiretroviral therapy: The ACTG (AIDS Clinical Trials Group) Study 5152s | | | | | | | | Prospective, multicenter study, Oct 2002 to Dec 2004  FU 24 weeks | | | | | 82 | | | USA | | | 54% white, 32% black or asian, 15% hispanic | | | 91 | | 35 (30-40) | | nd | | 245 (119-356) | | | nd | | 4.8(4.49-5.32) | | 25.1 (22.8-27.7) | | | | 44% | | 0% | | 33% PI sparing regimen, 33% NNRTI sparing regimen, 33% NRTI sparing regimen | | | 24 weeks | hsCRP | | | | no | = | nd |
|  |  | |  | | | | |  |  | |  | |  | | | |  | | | |  | | | | |  | |  | |  | | |  | | |  |  | | | | | |  | | |  | |  | | | | |  |  |  |  |
| Gleason,R L.,Jr;Caulk,AW.;Seifu,D; | | | Current Efavirenz (EFV) or Ritonavir-Boosted Lopinavir (LPV/r) Use Correlates with Elevate Markers of Atherosclerosis in HIV-Infected Subjects in Addis Ababa, Ethiopia | | | | | | | | Cross-sectional | | | | | HAART naive: 51 | | | Ethiopia | | | nd | | | 27 | | 38 (32-45) | | 1.6 (0.3-3.7) | | 395 (182-546) | | | nd | | 3.6 (2.2-4.7) | | 22 (20-26) | | | | 2% | | 81.9% | | 100% NRTI backbone, 66.2% NNRTI, 15.7% PI | | |  | hsCRP, sVCAM-1, sICAM-1 | | | | nd | = | ELISA |
|  |  |  |  |  |  |  |  |  |  |  |  |  |  |  |  | Efavirenz: 91 | | |  |  |  |  |  |  | 25 | | 38 (34-45) | | 5.7 (3.4-7.0) | | 349 (232-481) | | | | | <1.6(<1.6-<1.6) | | 21 (19-24) | | | | 0% | |  |  |  |  |  | 5.0 (3.2-6.2) |  |  |  |  |  |  |  |
|  |  |  |  |  |  |  |  |  |  |  |  |  |  |  |  | Nevarapine: 95 | | |  |  |  |  |  |  | 22 | | 37 (32-42) | | 6.0 (5.6-7.4) | | 390 (271-534) | | | | | <1.6(<1.6-<1.6) | | 23 (20-25) | | | | 2% | |  |  |  |  |  | 5.7 (3.9-6.2) |  |  |  |  |  |  |  |
|  |  |  |  |  |  |  |  |  |  |  |  |  |  |  |  | Lopinavir: 44 | | |  |  |  |  |  |  | 32 | | 39 (35-44) | | 6.5 (5.3-8.3) | | 285 (147-453) | | | | | <1.6(<1.6-<1.6) | | 22 (18-25) | | | | 0% | |  |  |  |  |  | 5.9 (4.2-6.8) |  |  |  |  |  |  |  |
| van Wijk,J. P.;de Koning,E. J.;Cabezas,M. C.; | | | Functional and structural markers of atherosclerosis in human immunodeficiency virus-infected patients | | | | | | | | Cross-sectional | | | | | Metabolic syndrome: 15 | | | Netherlands | | | nd | | | 100 | | 50 ± 3 | | 8.5 ± 0.8 | | 604 ± 105 | | | nd | | 1114 ± 824 copies/ml, 80% <50cp/mL | | 24.4 ± 0.5 | | | | 13% | | 100% | | 67% PI, 33% NNRTI, 100% NRTI | | | 4.8 ± 0.5 | hsCRP | | | | nd | unclear | high sensitivity kit: Quantex hs-CRP kit |
|  |  |  |  |  |  |  |  |  |  |  |  |  |  |  |  | No metabolic syndrome: 22 | | |  |  |  |  |  |  |  |  | 47 ± 2 | | 7.5 ± 5.2 | | 719 ± 58 | | |  |  | 813 ± 201 copies/ml, 77% <50cp/mL | | 23.6 ± 0.4 | | | | 14% | |  |  | 68% PI, 32% NNRTI, 100% NRTI | | | 4.2 ± 0.6 |  |  |  |  |  |  |  |
|  |  | |  | | | | |  |  | |  | |  | | | |  | | | |  | | | | |  | |  | |  | | |  | | |  |  | | | | | |  | | |  | |  | | | | |  |  |  |  |
| Ross Eckard,A.;Longenecker,C.;Jiang,Y | | | Lipoprotein-associated phospholipase A2 and cardiovascular disease risk in HIV infection | | | | | | | | Cross-sectional, March 2011 to Aug 2012 | | | | | 100 | | | USA | | | Caucasian 29%, Black 70%, other 1% | | | 77 | | 47 (25-68) | | 13 (1.5-26.8) | | 633 (142-1683) | | | 199 (0-614) | | 100% <1000 copies/mL. 80% undetectable | | 27 (7-17) | | | | 62% | | 100% | | 47 PI%, 52% NNRTI, 6% NRTI | | | 6.3 (0.8-21.7) | Lp-PLA2, Il-6, sTNRF-1, sTNFR-II, sVCAM-1, sICAM-1, d-dimer, fibrinogen, hsCRP, sCD14, sCD163. | | | | yes | = | ELISA, nephelometer (hsCRP, fibriongen), STA-R Coagualtion Analyzer (d-dimer) |
|  |  | | | | |  |  | | |  |  | | |  | | | |  | | | | |  | | | |  |  | |  | | | |  | |  | | | | |  | | |  | | |  | | | | |  |  |  |  |  |
| Sevastianova,K.;Sutinen,J.;Westerbacka,J.; | | | Arterial stiffness in HIV-infected patients receiving highly active antiretroviral therapy | | | | | | | | Cross-sectional | | | | | Lipodystrophy: 42 | | | Finland | | | 100% caucasian | | | 93 | | 44 ± 1 | | 8.6 ± 0.6 | | 567 ± 40 | | | 160 ± 20 | | 1.8 ± 0.1 | | 23.3 ± 0.4 | | | | 36 | | 100% | | 69% stavudine, 78% PI | | | 6.2 ± 0.4 | hsCRP | | | | no | = | high sensitivity commercial kit (Ultrasensitive CRP Kit) |
|  |  |  |  |  |  |  |  |  |  |  |  |  |  |  |  | No lipodystrophy: 17 | | |  |  |  |  |  |  | 76 | | 41 ± 2 | | 8 ± 1.2 | | 533 ± 60 | | | 210 ± 20 | | 1.5 ± 0.1 | | 23.1 ± 0.9 | | | | 35 | |  |  | 18% stavudine, 59% PI | | | 4.8 ± 0.8 |  |  |  |  |  |  |  |
|  |  | |  | | | | |  |  | |  | |  | | | |  | | | |  | | | | |  | |  | |  | | |  | | |  |  | | | | | |  | | |  | |  | | | | |  |  |  |  |
| Jang,J. J.;Schwarcz,A. I.;Amaez,D. A | | | Elevated osteoprotegerin is associated with abnormal ankle brachial indices in patients infected with HIV: a cross-sectional study | | | | | | | | Cross-sectional, Dec 2005 to May 2006 | | | | | 102 | | | USA | | | 45% Hispanic, 44% African American, <1% Asian | | | 57 | | 48.4 ± 9.1 | | 141.1 ± 63.6 months | | 565.4 ± 416.3 | | | nd | | 10,856.5 ± 47,682.8 copies/mL | | 27.4 ± 6.0 | | | | 75% ever smokers | | nd | | nd | | | 52.7 ± 56.6 months | CRP, IL-1B, IL-6, OPG | | | | nd | ? | IMMAGE 800 assay for CRP and ELISA for IL-1B, IL-6 and OPG |
|  | |  | | |  | | |  | |  | |  | | |  | | | | | | |  | |  | | |  |  | | | |  | | |  | | | | |  | |  | | |  | | | | | |  |  |  |  |  |  |
| Kaplan,R. C.;Sinclair,E.;Landay,A. L.; | | | T cell activation predicts carotid artery stiffness among HIV-infected women | | | | | | | | Cross-sectional. Substudy of Women's Interagency HIV study. | | | | | 114 | | | USA | | | White/other 9%, hispanic 27%, African American 64% | | | - | | 46 (43-50) | | nd | | 384 (227-582) | | | 210 (109-339) | | 1.3 (0.08-16) (thousands) | | 32% <25, 39% 25-30, 29% >30 | | | | 48% | | 64% | | 63% NRTI, 24% NNRTI, 27% 1 PI, 18% 2 PI's | | | nd | CRP | | | | nd | several months | nephelometry |
| **18FDG PET** | | |  | | | | | | | |  | | | | |  | | |  | | |  | | |  | |  | |  | |  | | |  | |  | |  | | | |  | |  | |  | | |  |  | | | |  |  |  |
| Knudsen,A;Hag,A;Loft,A | | | HIV infection and arterial inflammation assessed by (18)F-fluorodeoxyglucose (FDG) positron emission tomography (PET): a prospective cross-sectional study | | | | | | | | Cross-sectional, March 2011 to June 2013 | | | | | 26 | | | Denmark | | | nd | | | 100 | | 50.5 ± 2.4 | | 13.9 (10.8-16.8) | | 636 (549-717) | | | nd | | 19 (19-31) copies/mL | | 24.0 (22.9-25.1) | | | | 19% | | 100% | | ≥2 NRTIs + 1 NNRTI (81%), ≥2 NRTIs + ≥1 PI (15%), other 4% | | | 9.9 (8-11.7) | hsCRP, sCD163, sE-selectin, sVCAM-1, sICAM-1, MMP-9, PAI-1 | | | | nd | = | Siemens STRATUS CS (hsCRP), ELISA (sCD163), multiplex assay (sE-selectin, sVCAM-1, sICAM-1, MMP-9 and PAI-1) |
| **18FDG PET and CAC** | | | | | | | | | | |  | | | | |  | | |  | | |  | | |  | |  | |  | |  | | |  | |  | |  | | | |  | |  | |  | | |  |  | | | |  |  |  |
| Subramanian,S.;Tawakol,A.;Burdo,T. | | | Arterial inflammation in patients with HIV | | | | | | | | Cross-sectional, Nov 2009 to July 2011 | | | | | 27 | | | USA | | | nd | | | 93 | | 51.6 (49.5-53.6) | | 15.5 ± 5.7 | | 641 ± 288 | | | 99 (50, 250) | | <48 (<48-<48) 81% <48 copies/mL | | nd | | | | 22% | | 100% | | 41% PI, 96% NRTI, 52% NNRTI | | | 12.3 ± 4.3 | hsCRP, D-dimer, sCD163 | | | | nd | nd | hsCRP: immunochemiluminometric assay. sCD163: ELISA. D-dimer: immunoturidimetric assay |
| **MRI of thoracic aorta and carotid arteries** | | | | | | | | | | |  | | | | |  | | |  | | |  | | |  | |  | |  | |  | | |  | |  | |  | | | |  | |  | |  | | |  |  | | | |  |  |  |
| Floris-Moore,M.;Fayad,Z. A.;Berman,J. W.; | | | Association of HIV viral load with monocyte chemoattractant protein-1 and atherosclerosis burden measured by magnetic resonance imaging | | | | | | | | Cross sectional | | | | | viral load <75 copies/mL: 38 | | | USA | | | black 65.8%, latino 28.9%, white/other 5.3% | | | 55 | | 54.7 ± 1.0 | | nd | | <200: 7.9%, 200-499: 44.7%, >500 47.4% | | | nd | | 61% detectable viremia | | 25.2 ± 0.7 | | | | 47.4% | | 84.2% | | 57.9% PI based, 26.3% non-PI | | | PI users: median 66 months (42-98) | MCP-1, CCL2 | | | | yes | = | ELISA |
|  |  |  |  |  |  |  |  |  |  |  |  |  |  |  |  | viral load ≥75 copies/mL: 60 | | |  |  |  | black 70%, latino 15%, white/other 15% | | | 65 | | 54.9 ± 0.7 | |  |  | <200: 25.0%, 200-499: 46.7%, >500 28.3% | | | nd | |  |  | 26.9 ± 0.8 | | | | 68.3% | | 56.7% | | 41.7% PI based, 15.0% non-PI | | |  |  |  |  |  |  |  |  |
|  |  | | | | |  |  | | |  |  | | |  | | | |  | | | | |  | | | |  |  | |  | | | |  | |  | | | | |  | | |  | | |  | | | | |  |  |  |  |  |
| Mariano-Goulart,D.;Jacquet,J. M.;Molinari,N | | | Should HIV-infected patients be screened for silent myocardial ischaemia using gated myocardial perfusion SPECT? | | | | | | | | Cross-sectional, Nov 2009 to Jan 2012 | | | | | 94 | | | France | | | nd | | | 87 | | 55 ± 8 | | 16 ± 7 | | 555 ± 261 | | | nd | | 19% >20cp/mL | | 24 ± 4 | | | | 64% | | nd | | NRTI 89%, NNRTI 33%, Integrase Inhibitor 14%, PI 67% | | | 12 ± 6 | CRP | | | | nd | nd | nd |
|  | | | |  | | | |  | |  |  | | | | |  | | | |  | | | | | | |  | |  | |  | | | | | | | |  |  |  |  |  |  |  |  |  |  |  |  |  |  |  |  |  |  |
| Kristoffersen,U. S.;Lebech,A. M.;Wiinberg,N.; | | | Silent ischemic heart disease and pericardial fat volume in HIV-infected patients: a case-control myocardial perfusion scintigraphy study | | | | | | | | Cross-sectional, Sept 2008 to July 2010 | | | | | 105 | | | Denmark | | | nd | | | 89 | | 47.4 ± 0.83 | | 12.3 ± 0.64 | | 636 ± 25 | | | 171 ± 11 | | 90 % < 40 copies/mL | | 24.7 ± 0.33 | | | | 37% | | 100% | | ≥2 NRTIs + 1 NNRTI (62%), ≥2 NRTIs + ≥1 PI (24%), NRTI + NNRTI + PI (6%), other (9%) | | | 8.9 ± 0.41 | sICAM-1, sVCAM-1, MMP-9, tPAI-1, hsCRP, endothelin. | | | | nd | = | Fluorescent bead-based immunoassay (sICAM-1, sVCAM-1,MMP9, hsCRP, tPAI-1). ELISA (Endothelin) |
