# Supplementary material for: Association between Immune Markers and Surrogate Markers of Cardiovascular Disease in HIV Positive Patients: A Systematic Review
Source: PLoS One. 2017 Jan 13;12(1):e0169986. doi: 10.1371/journal.pone.0169986 (PMC5234789; doi:10.1371/journal.pone.0169986)
Supplement: S2 Table — ND: no data. (DOCX) [file pone.0169986.s003.docx]

| \| **Author** \| \| --- \| | **Title** | **Clear inclusion criteria** | **Homogenous moment of inclusion/how were patients included** | **Standardized: determinant** | **Standardized: outcome** | **Missing data with regard to inclusion or follow-up** | **Blinded measurement of determinant / outcome** | **Outcome** | **Adjustment for confounders** |
| --- | --- | --- | --- | --- | --- | --- | --- | --- | --- | --- |
| **CT coronary angiography and coronary artery calcium score (CAC)** | |  | | | | | | | |
| Burdo,T. H.;Lo,J.;Abbara,S.; | Soluble CD163, a novel marker of activated macrophages, is elevated and associated with noncalcified coronary plaque in HIV-infected patients | yes | HIV+ men recruited from HIV clinics, community health care centers and newspaper advertisements. Aged 18-58 years without known cardiac diseaese or symptoms suggestive of cardiac disease. Excl: known renal disease or creat levels >1.5 mg/dL or estimated GFR <70. Patients on ART were required to have been on stable therapy for >3 months. | yes | yes | nd on potential eligible pts | Outcome | Coronary plaque (calcified/non-calcified) on CT coronary angiography, based on consensusreading by 2 investigators, and CAC (agatson). Severe coronary artery stenosis was defined as luminal obstruction 70% diameter | age, race, lipids, blood pressure, glucose, smoking, HIV infection, lipid lowering therapy, sCD14 |
| D'Ettorre,G.;Ceccarelli,G.;Francone,M.; | High prevalence of coronary stenosis detected by Coronary CT angiography in asymptomatic HIV-infected subjects with low cardiovascular risk | yes | Asymptomatic HIV+ patients referred by physicians with a low cardiovascular risk, no HCV co-infection. | yes | yes | nd on potential eligible pts | No | Coronary plaque on CT coronary angiography. Read independently by two radiologists. Luminal narrowing >50%: clinicallsy significant coronary stenosis, than: coronary angiography. Coronary calcium score (agatson). | not clear if CRP was used in multivariable analysis. Only univariate data |
| Fitch,Kv;Srinivasa,S.;Abbara,S.; | Noncalcified coronary atherosclerotic plaque and immune activation in HIV-infected women | yes | HIV+ women recruited from HIV clinics, community health centers and newspaper advertisements. Aged 18-60 years, without symptoms or history of CVD, stable ART >3 months. | yes | yes | nd on potential eligible pts | Outcome | Coronary plaque (calcified/non-calcified) on CT coronary angiography, based on consensusreading by 2 investigators, and CAC (agatson). Severe coronary artery stenosis was defined as luminal obstruction 70% diameter | No |
| Hwang,J. J.;Wei,J.;Abbara,S.; | Receptor activator of nuclear factor-kappaB ligand (RANKL) and its relationship to coronary atherosclerosis in HIV patients | yes | Substudy of SATURN-HIV trial. 18-55 years, without symptoms or history of cardiac disease. In case of ART use stable >3 months. | yes | yes | nd on potential eligible pts | Outcome | Coronary plaque on CT coronary angiography and CAC (agatson) | Framingham risk score, HIV related factors |
| Lai,S.;Bartlett,J.;Lai,H.; | Long-term combination antiretroviral therapy is associated with the risk of coronary plaques in African Americans with HIV infection | yes | HIV infected patients, consecutively enrolled in a hospital, 25-54 years, African American race without hypertension or cardiac disease | ND | yes | nd on potential eligible pts | Outcome | MDCT for CAC and CT coronary angiography | Univariable analysis. Factors with p ≤0.10 were included in multivariable analysis |
| Lo,J.;Abbara,S.;Shturman,L. | Increased prevalence of subclinical coronary atherosclerosis detected by coronary computed tomography angiography in HIV-infected men | yes | HIV infected patients recruited from HIV clinics and ads in newspapers, aged 18-55 years, without (symptoms of) CVD. In case of ART stable >3 months | yes | yes | nd on potential eligible pts | Outcome | Coronary plaque on CT coronary angiography. CAC (agatson) | HIV related parameters and cardiovascular risk factors |
| McKibben,R A.;Margolick,J B.;Grinspoon,S; | Elevated levels of monocyte activation markers are associated with subclinical atherosclerosis in men with and those without HIV infection | yes | Substudy of MACS. HIV infected homosexual patients, aged 40-70 years, no prior cardial intervention. | yes | yes | yes >10% | Outcome | Coronary plaque on noncontrast cardiac CT, CAC (agatson). Moderate stenosis 50-69%, severe stenosis ≥70%. | CVD risk factors, age, race, HIV-associated factors |
| **Coronary calcium score (CAC)** | | | | | | | | | |
| Jang,J. J.;Berkheimer,S. B.;Merchant,M.; | Asymmetric dimethylarginine and coronary artery calcium scores are increased in patients infected with human immunodeficiency virus | no | HIV+ patients ≥18 years and controls matched for age and gender. | yes | yes | ND | ND | CAC | HIV status, creatinine and HDL |
| Longenecker,C. T.;Jiang,Y.;Orringer,C. E.; | Soluble CD14 is independently associated with coronary calcification and extent of subclinical vascular disease in treated HIV infection | yes | Substudy of SATURN-HIV trial, aged ≥18 years, stable ART ≥12 weeks, HIV RNA <1000 cp/mL, hs-CRP≥2mg/l, no coronary disease or diabetes. | yes | yes | nd on potential eligible pts | ND | CAC, FMD (CAC present : >5 pixels >130HU) | age, sex, race, nadir CD4+ cell count, LDL-C, limb fat |
| Mangili,A.;Ahmad,R.;Wolfert,R. L. | Lipoprotein-associated phospholipase A2, a novel cardiovascular inflammatory marker, in HIV-infected patients | yes | Substudy within the CARE trial. HIV-infected patients without diabetes, uncontrolled hypertension , myocardial infarction or stroke < 6 months. | yes | yes | nd on potential eligible pts | ND | CAC. CAC stratified into 0, 1-100, >100. | standard risk factors |
| Shikuma,C. M.;Barbour,J. D.;Ndhlovu,L. C.; | Plasma monocyte chemoattractant protein-1 and tumor necrosis factor-(alpha) levels predict the presence of coronary artery calcium in HIV-infected individuals independent of traditional cardiovascular risk factors | yes | Substudy of HAHC-CVD study, patients baseline data used. Inclusion: HIV infected patients on stable ART≥6months, ≥40yrs. | yes | unclear | nd on potential eligible pts. 7/130 patients have partially missing data. | Outcome | CAC. CAC present in case of Agatston score >0. | If univariable significant, multivariable adjusted for age, gender, CD4 percent, hypertension, diabetes, smoking history, total cholesterol/HDL ratio. |
| **Flow Mediated Dilation (FMD)** | | | | | | | | | |
| Gupta,S. K.;Mi,D.;Dube,M. P.; | Pentoxifylline, inflammation, and endothelial function in HIV-infected persons: a randomized, placebo-controlled trial | yes | Patients with HIV not requiring ART per DHHS Guidelines, aged ≥18yrs, CD4 cell count≥350uL at screening. Excl: known cardiovascular disease and risk factors of CVD | yes | yes | yes >10% | Outcome | FMD and nitroglycerin-mediated dilation (NTGMD) | correlation |
| Hileman,C. O.;Longenecker,C. T.;Carman,T. L.; | Elevated D-dimer is independently associated with endothelial dysfunction: a cross-sectional study in HIV-infected adults on antiretroviral therapy | yes | HIV infected, stable ART >3months, HIV-1 RNA <400cp/mL, FMD measurement available. Part of a study at the HIV Metabolic Research Center | yes | yes | nd on potential eligible pts | ND | FMD | age, sex, race, BMI, CD4+ T-cell count, whether on a protease inhibitor and smoking status and all variables with P<0,25 in univariable analysis |
| Masia,M.;Padilla,S.;Garcia,N.; | Endothelial function is impaired in HIV-infected patients with lipodystrophy | yes | Consecutive healthy HIV adults with lipodystrophy if ART >2 years, stable >6months, random control subject (1:1) without lipodystrophy mached by age (±5 years) and sex. | yes | yes | yes >10% in potential eligible patients | Yes | FMD | traditional CVD risk factors, pro-atherosclerotic biomarkers and factors associated with HIV infection |
| Nolan,D.;Watts,G. F.;Herrmann,S. E.; | Endothelial function in HIV-infected patients receiving protease inhibitor therapy: does immune competence affect cardiovascular risk? | yes | Subgroup of the Western Australian HIV cohort. Incl: HIV+ men, referred to lipid disorder clinic, PI >9 monhts. | yes | yes | nd on potential eligible pts | Outcome | FMD | smoking status, BMI, lipid and lipoprotein levels, fastin insulin, HOMA-R duration of PI therapy, age, mean arterial pressure, baseline arterial diameter, pulse pressure, %CD4 T cell count. |
| Solages,A.;Vita,J. A.;Thornton,D. J. | Endothelial function in HIV-infected persons | yes | HIV+ from a cohort of Hep C infected patients. Excl: hemodialysis, uncontrolled hypertension | ND | yes | nd on potential eligible pts. 1/76 patients with missing data. | Outcome | FMD | Age, BMI, smoking, total cholesterol, fasting blood glucose, seks and factors with P≤0,15 in univariate analysis |
| Stein,Jh;Brown,Tt;Ribaudo,Hj; | Ultrasonographic measures of cardiovascular disease risk in antiretroviral treatment-naive individuals with HIV infection | yes | Baseline evaluation of ART naive HIV infected patients enrolled in a randomized ART treatment trial (AIDS clinical trials group study A5257). Age ≥18 yrs, HIV RNA >1000cp/mL. Excl: known CVD | yes | yes | nd on potential eligible pts | Outcome | FMD | selection of variables based on Akaiki Information Criteria. For the final model selection was done based on clinical input, colllinearity, final model r2 values. |
| Torriani,F. J.;Komarow,L.;Parker,R. A. | Endothelial function in human immunodeficiency virus-infected antiretroviral-naive subjects before and after starting potent antiretroviral therapy: The ACTG (AIDS Clinical Trials Group) Study 5152s | yes | Substudy of ACTG5142, subjects recruited consecutively from six sites.Incl: HIV+, HIV RNA >2.0 log10 cp/mL. Excl: o.a. CVD | ND | yes | <10% on baseline, >10% at 24 weeks follow up. Nd on potential eligible pts | Outcome | FMD | correlation |
| **Flow mediated dilation (FMD) and pulse wave velocity (PWV)** | |  |  |  |  |  |  |  |  |
| Gleason,R L.,Jr;Caulk,AW.;Seifu,D | Current Efavirenz (EFV) or Ritonavir-Boosted Lopinavir (LPV/r) Use Correlates with Elevate Markers of Atherosclerosis in HIV-Infected Subjects in Addis Ababa, Ethiopia | yes | HIV+ and HIV- patients recruited from a referral hospital, 18-65 years, on the same ART regimen >2 months. No AIDS defining illnesses or diabetes mellitus | yes | yes | No data on potential eligible patients. Biomarkers mssing in >10% of subjects | Outcome | PWV and FMD | Relevant study parameters or p <0.05 in correlation analysis |
| van Wijk,J. P.;de Koning,E. J.;Cabezas,M. C. | Functional and structural markers of atherosclerosis in human immunodeficiency virus-infected patients | yes | HIV infected men, 18-70 years, recruited from the Department of Infectious Diseases. HIV-RNA <10.000cp/mL, HAART ≥12 months. | yes | yes | nd on potential eligible pts | ND | PWV and FMD | Correlation, significant variables were enterd in regression analysis |
| **Flow mediated dilation (FMD) and coronary calcium score (CAC)** | |  |  |  |  |  |  |  |  |
| Ross Eckard,A.;Longenecker,C.;Jiang,Y.; | Lipoprotein-associated phospholipase A2 and cardiovascular disease risk in HIV infection | yes | First 100 subjects. HIV+, age≥18yars, LDL≤130mg/dL, ART≥ 6 months, stable ≥ 3 months, HIV-1 RNA <1000cp/mL, no CVD or statin use | yes | yes | no | Outcome | CAC, FMD | only correlation for analysis of interest |
| **Pulse wave analysis (PWA)** |  |  |  |  |  |  |  |  |  |
| Sevastianova,K.;Sutinen,J.;Westerbacka,J.; | Arterial stiffness in HIV-infected patients receiving highly active antiretroviral therapy | yes | HIV patients recruited from HIV outpatient clinic. HAART ≥18 months, stable ≥3 months | yes | yes | nd on potential eligible pts | ND | PWA | All baseline variables with a p-value ≤0.20 were enterd in regression analysis |
| **Ankle brachial index (ABI)** |  |  |  |  |  |  |  |  |  |
| Jang,J. J.;Schwarcz,A. I.;Amaez,D. A.; | Elevated osteoprotegerin is associated with abnormal ankle brachial indices in patients infected with HIV: a cross-sectional study | yes | HIV-infected patients ≥18years recruited from primary HIV clinic on consecutive clinic days. No vascular disease of non-atherosclerotic origin. | yes | yes | nd on potential eligible pts | ND | ABI. Definite peripheral arterial disease: ABI ≤0.90 | age, sex, BMI, smoking, diabetes mellitus, total cholesterol, HDL, low densitiy lipoprotein, triglycerides, CRP, cardiovascular disease, family cardiac history, duration of HIV and duration of PI use. |
| **Carotid artery stiffness (carotid artery distensibility and Young's elastic modulus)** | |  |  |  |  |  |  |  |  |
| Kaplan,R. C.;Sinclair,E.;Landay,A. L. | T cell activation predicts carotid artery stiffness among HIV-infected women | no | Substudy within WIHS (enrollment 1994-1995 and 2001-2002). Random sample of women aged≥40yrs, without CVD, with available carotid ultrasounds within several months of collection of peripheral blood cells. | yes | yes | nd on potential eligible pts | ND | Carotid arterial stiffness (distensibility, Young's elastic modulus) | age, cardiovascular risk factors, HIV RNA, CD4+ T-cell count |
| **18FDG PET** |  |  |  |  |  |  |  |  |  |
| Knudsen,A;Hag,A;Loft,A | HIV infection and arterial inflammation assessed by (18)F-fluorodeoxyglucose (FDG) positron emission tomography (PET): a prospective cross-sectional study | yes | Recruited from routine visits out-patient clinic department infectious diseases. Incl: HIV+, men, age ≥18 years, ART >12 months, no CVD. | yes | yes | nd on potential eligible pts | No | 18FDG PET/CT with concominant measurement of intima-media thickness | Only the marker that was significantly different between HIV+ and HIV- was analyzed in multiple regression. |
| Subramanian,S.;Tawakol,A.;Burdo,T. H.; | Arterial inflammation in patients with HIV | yes | Prospectively enrolled HIV+ patiens without known CVD, stable ART ≥3 months. Unclear were cases were recruited. | yes | yes | nd on potential eligible pts | Outcome | 18FDG PET/CT, CAC | Correlation |
| **MRI of thoracic aorta and carotid arteries** |  |  |  |  |  |  |  |  |  |
| Floris-Moore,M.;Fayad,Z. A.;Berman,J. W. | Association of HIV viral load with monocyte chemoattractant protein-1 and atherosclerosis burden measured by magnetic resonance imaging | yes | Participants with HIV with CIMT ≥ 0.7mm, ≥45 years, not on lipid lowering drugs. | yes | yes | yes | Outcome | MRI of thoracic aorta and carotid arteries | age, BMI, current cigarette smoking. |
| **Myocardial perfusion scintigraphy SPECT** |  |  |  |  |  |  |  |  |  |
| Mariano-Goulart,D.;Jacquet,J. M.;Molinari,N.; | Should HIV-infected patients be screened for silent myocardial ischaemia using gated myocardial perfusion SPECT? | yes | HIV+ patients with CV risk factors without signs of CVD after cardiac examination. Prospectively recruited from hospital (outpatient). | ND | yes | yes, 5 of 99 recruited patients not included. ND on potential eligible pts | ND | Myocardial SPECT. Ischemia: reversible perfusion defect and necrosis as a fixed significant defect with abnormal wall thickening | values with p< 0.25 were tested in multivariable analysis |
| **Myocardial perfusion scintigraphy (MPS), CAC, pericardial fat volume measurement (PFVM)** | |  |  |  |  |  |  |  |  |
| Kristoffersen,U. S.;Lebech,A. M.;Wiinberg,N.; | Silent ischemic heart disease and pericardial fat volume in HIV-infected patients: a case-control myocardial perfusion scintigraphy study | yes | Consecutive HIV patients, age 18-70 years, receiving cART >12 months, prospectively recruited at routine visits at outpatient clinic. | yes | yes | nd on potential eligible pts | Outcome | Myocardial perfusion scintigraphy, coronary artery calcium score, pericardial fat volume measurement, carotid intima media thickness | metabolic syndrome, smoking status, age, gender, cholesterol, triglycerides, glucose and systolic blood pressure |
